# *N*-n-Butyl Haloperidol Iodide Ameliorates Oxidative Stress in Mitochondria Induced by Hypoxia/Reoxygenation through the Mitochondrial c-Jun N-Terminal Kinase/Sab/Src/Reactive Oxygen Species Pathway in H9c2 Cells

**DOI:** 10.1155/2019/7417561

**Published:** 2019-05-08

**Authors:** Qianwen Chu, Yanmei Zhang, Shuping Zhong, Fenfei Gao, Yicun Chen, Bin Wang, Zhaojing Zhang, Wenfeng Cai, Weiqiu Li, Fuchun Zheng, Ganggang Shi

**Affiliations:** ^1^Department of Pharmacy, Jiading District Central Hospital Affiliated Shanghai University of Medicine & Health Sciences, Shanghai 201800, China; ^2^Department of Pharmacology, Shantou University Medical College, Shantou 515041, China; ^3^Pharmaceutical Laboratory, The First Affiliated Hospital, Shantou University Medical College, Shantou 515041, China; ^4^Department of Medicine, Keck School of Medicine, University of Southern California, Los Angeles, California 90033, USA; ^5^Department of Medical Genetics and Cell Biology, School of Basic Medical Sciences, Zhengzhou University, Zhengzhou 450003, China; ^6^Analytical Cytology Laboratory, Shantou University Medical College, Shantou 515041, China; ^7^Clinical Pharmacology Laboratory, The First Affiliated Hospital, Shantou University Medical College, Shantou 515041, China

## Abstract

Both c-Jun N-terminal kinase (JNK) and reactive oxygen species (ROS) play important roles in myocardial ischemia/reperfusion (I/R) injury. Our previous studies suggest that *N*-n-butyl haloperidol iodide (F_2_) exerts cardioprotection by reducing ROS production and JNK activation caused by I/R. In this study, we hypothesized that there is a JNK/Sab/Src/ROS pathway in the mitochondria in H9c2 cells following hypoxia/reoxygenation (H/R) that induces oxidative stress in the mitochondria and that F_2_ exerts mitochondrial protective effects during H/R injury by modulating this pathway. The results showed that H/R induced higher-level ROS in the cytoplasm on the one hand and JNK activation and translocation to the mitochondria by colocalization with Sab on the other. Moreover, H/R resulted in mitochondrial Src dephosphorylation, and subsequently, oxidative stress evidenced by the increase in ROS generation and oxidized cardiolipin in the mitochondrial membranes and by the decrease in mitochondrial superoxide dismutase activity and membrane potential. Furthermore, treatment with a JNK inhibitor or Sab small interfering RNA inhibited the mitochondrial translocation of p-JNK, decreased colocalization of p-JNK and Sab on the mitochondria, and reduced Src dephosphorylation and mitochondrial oxidative stress during H/R. In addition, Src dephosphorylation by inhibitor PP2 increased mitochondrial ROS production. F_2_, like inhibitors of the JNK/Sab/Src/ROS pathway, downregulated the H/R-induced mitochondrial translocation of p-JNK and the colocalization of p-JNK and Sab on the mitochondria, increased Src phosphorylation, and alleviated the above-mentioned mitochondrial oxidative stress. In conclusion, F_2_ could ameliorate H/R-associated oxidative stress in mitochondria in H9c2 cells through the mitochondrial JNK/Sab/Src/ROS pathway.

## 1. Introduction

Ischemia/reperfusion (I/R) injury refers to an increase in organic damage that occurs with the restoration of blood flow after ischemia. Myocardial I/R injury is considered a major health threat and is associated with acute coronary syndromes. Timely coronary revascularization has become a routine treatment approach for patients with significant ST-segment elevation, but reperfusion of blood into the previous ischemic area is always accompanied by increased myocardial injury. Studies have shown that a small amount of reactive oxygen species (ROS) has cardioprotective effects [1]. For example, redox signaling derived from ROS is responsible for cardiomyocyte differentiation and excitation-contraction coupling [2]. Wu et al. [3] also demonstrated that during early reperfusion, the moderate ROS plays an important role in the intermittent hypobaric hypoxia-afforded cardioprotection by activating the JAK2/STAT3 pathway against I/R-induced Ca^2+^ overload and contractile dysfunction. On the contrary, a large number of studies have shown that overgenerated ROS play a vital role in the pathological process of myocardial I/R injury, which not only induces oxidative stress injury but also participates in other I/R-associated damages [4]. As our previous studies show, ROS accumulation leads to oxidation of the lipid structure of the cell membrane, increases cell membrane permeability, and subsequently causes the leakages of CK, LDH, and cTnI from cardiomyocyte [5, 6] in hypoxia/reoxygenation (H/R). Overproduced ROS during I/R is responsible for the damage of mitochondrial DNA [7], protein oxidation [8], inflammation [9], apoptosis [10], and necrosis. However, the specific pathogenesis about the relationship between ROS and myocardial I/R injury remains obscure.

The mitochondrion is an important organelle that is responsible for ROS production, oxidative phosphorylation, energy metabolism, and maintenance of calcium homeostasis. In previous studies, mitochondria have been shown to be the main site of ROS generation and a key target for cardiac protection strategies during reperfusion. Mitochondrial ROS are derived from mitochondrial respiratory chain and ROS production enzymes. When the myocardium suffers from I/R, a burst of ROS generated from the mitochondria will trigger mitochondrial permeability transition pore opening, decrease myocardial contractile function, induce arrhythmia, and prompt myocardial apoptosis or necrosis.

The c-Jun N-terminal kinase (JNK) (known as stress-activated protein kinases) is a key modulator in cell death mediated by ROS [11]. In our previous work, we found that ROS can promote the phosphorylation of JNK in the cytoplasm and cause H/R injury when cardiac microvascular endothelial cells and H9c2 cells are subjected to H/R [5, 12]. Proto-oncogene tyrosine-protein kinase Src (c-Src) was the first tyrosine kinase to be identified [13, 14]. In a mechanism study of ischemic preconditioning, Ge et al. found that mitochondrial Src protein regulates the mitochondrial complex I activity and regulates mitochondrial ROS production to protect the myocardium from I/R injury [15]. Recently, Win et al. [16] found that activated JNK could bind to the mitochondrial outer membrane SH3 domain-binding protein 5 (SH3BP5 or Sab), leading to SHP1 protein bind to Sab protein release to the mitochondrial inner membrane, which ultimately resulted in SHP1-dependent inactivation of p-Src in liver injury models. Chambers et al. [17] have also reported that H_2_O_2_/iron sulfate (FeSO_4_) can activate JNK signaling and cause it to translocate to the mitochondria via Sab in H9c2 cells. Taken together, we speculated that a JNK/Sab/Src/ROS signaling pathway triggered by cytoplasmic ROS may exist in mitochondria during myocardial I/R and that this “ROS-induced ROS release” will at first give rise to mitochondrial oxidative stress and then to whole-cell oxidative stress.

*N*-n-Butyl haloperidol iodide (F_2_) (Chinese national invention patent no. ZL96119098.1) synthesized by our laboratory is a new type of calcium antagonist derived from haloperidol, which is used to treat psychological diseases. Previous studies have confirmed that F_2_ could protect cardiomyocytes from I/R injury in vivo or H/R injury in vitro. The mechanism of F_2_ may be related to blocking of the cell membrane Ca^2+^ channel and the inhibition of Egr-1 overexpression, which has been well documented to be closely associated with I/R-mediated inflammation [6, 18, 19]. It also has been found that F_2_ can improve intracellular oxidative stress [5, 12], such as decreasing the level of ROS, increasing the activity of superoxide dismutase (SOD), and reducing the content of malonaldehyde (MDA). Moreover, studies have demonstrated that F_2_ inhibits cytoplasmic JNK activation in cardiomyocytes during H/R and that cytoplasmic ROS mediates the process.

In this study, we constructed an H/R injury model of H9c2 cells by using a hypoxia/anaerobic workstation to simulate myocardial I/R injury and observed whether a JNK/Sab/Src/ROS signaling pathway was present in the mitochondria and could trigger mitochondrial oxidative stress. On the basis of this, we aimed to investigate whether F_2_ ameliorates mitochondrial oxidative stress injury during H/R by regulating the above pathway.

## 2. Materials and Methods

### 2.1. Reagent Preparation

The H9c2 cell line was from the American Type Culture Collection (ATCC, Rockville, MD, USA). F_2_ was synthesized by our laboratory (Chinese national invention patent no. ZL96119098.1). Dulbecco's modified Eagle's medium (DMEM) and fetal bovine serum (FBS) were obtained from Gibco Laboratories (Carlsbad, CA, USA). The JNK inhibitor SP600125 was from Enzo Life Sciences (Farmingdale, NY, USA). The JNK activator anisomycin and 2,7′-dichlorofuorescein acetyl acetate were obtained from Sigma-Aldrich (St. Louis, MO, USA). Small interfering ribonucleic acids (siRNAs) were purchased from Shanghai GenePharma Co. Ltd. (Suzhou, China). The 3-(4,5-dimethyl-2-thiazolyl)-2,5-diphenyl-2-H-tetrazolium (MTT) reagent was gathered from Amresco (Solon, OH, USA). A mitochondria isolation kit and JC-1 dye kit were obtained from the Beyotime Institute of Biotechnology (Shanghai, China). A Pierce™ Bicinchoninic Acid Assay Kit and SuperSignal detection kit were obtained from Thermo-Fisher Scientific (Waltham, MA, USA), as were Alexa Fluor™ 594-conjugated anti-mouse secondary antibody, Alexa Fluor™ 488-conjugated anti-rabbit antibody, and mitochondria-selective dye MitoSOX™ Red.

The primary antibodies used for Western blot analysis in this study were as follows: p-JNK (no. 4668), JNK (no. 9252), p-Src (Tyr416) (no. 6943), c-Src (no. 2109), and COX-IV (no. 4850) (Cell Signaling Technology, Danvers, MA, USA); Sab (H00009467-M01 from Novus Biologicals, Littleton, CO, USA); and *β*-actin (no. 66009-1-Ig from the Proteintech Group, Chicago, IL, USA). The secondary antibody against *β*-actin, anti-rabbit, and anti-mouse were purchased from Wuhan Boster Bio-Engineering Ltd. Co. (Wuhan, China).

### 2.2. H9c2 Cell Culture and Hypoxia/Reoxygenation Protocol

H9c2 cells were cultured in DMEM supplemented with 10% FBS at 37°C under 5% CO_2_. To induce hypoxia stress, H9c2 cells were cultured in hypoxia buffer solution [20] (137 mM of sodium chloride, 12 mM of potassium chloride, 0.49 mM of magnesium dichloride hexahydrate, 0.9 mM of calcium chloride, 4 mM of 4-(2-hydroxyethyl)-1-piperazineethanesulfonic acid, and 20 mM of sodium lactate, add hydrogen chloride to pH 6.2) and cultured in a hypoxia/anaerobic workstation (Ruskinn, England) (1% O_2_, 5% CO_2_, 94% N_2_, 37°C) for 1, 2, 4, 6, or 8 hours. Then, the hypoxia buffer solution was replaced with fresh culture medium containing 10% FBS, and the cells were transferred into a normoxic incubator for 1 hour of reoxygenation.

### 2.3. Sab Small Interfering RNA

The protocol of Sab small interfering RNA is similar to our previous study [21]. The cells were inoculated in 6-well cell culture dishes and transfected with Sab siRNA by Gibco™ Opti-MEM™ media (Thermo-Fisher Scientific, Waltham, MA, USA). In brief, 3 *μ*l of 20 *μ*M Sab siRNA-1, 3 *μ*l of 20 *μ*M Sab siRNA-2, and 4 *μ*l of 20 *μ*M Sab siRNA-3—that is, Sab siRNA-1 : Sab siRNA-2 : Sab siRNA-3 = 3 : 3 : 4—or 10 *μ*l negative control (NC) siRNA were mixed with 250 *μ*l of the Opti-MEM™ media, separately (the sequences of each siRNA and negative control were shown in Table 1). Next, Lipofectamine™ 2000 (Invitrogen, Carlsbad, CA, USA) was mixed with the Opti-MEM™ media, and then, the mixtures were combined for 20 minutes at room temperature. Subsequently, H9c2 cells were incubated with mixture for 6 hours. At last, the medium was then changed into antibiotic-free DMEM supplemented with 10% FBS for 48 hours.

### 2.4. Experimental Groups

**Figure 1:**
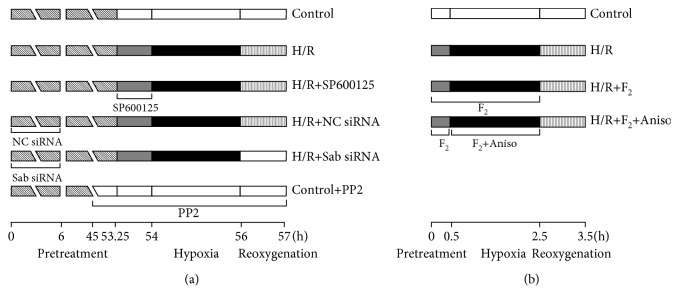
The protocol of experimental grouping and treatments: (a) protocol used in investigating whether the mitochondrial JNK/Sab/Src/ROS signaling pathway is activated in H9c2 cells after H/R and (b) protocol used in investigating the role of F_2_ in H9c2 cells after H/R.

H9c2 cells were randomly allocated to the following groups: control, H/R, H/R+JNK inhibitor (H/R+SP600125), H/R+negative control siRNA (H/R+NC siRNA), H/R+Sab siRNA, control+Src inhibitor (control+PP2), H/R+F_2_, and H/R+F_2_+JNK agonist (H/R+F_2_+Aniso). The working concentration and the time of each group were as follows: the cultured solution of the control group is replaced with fresh DMEM before the experiment, and 20 *μ*M SP600125 was preincubated for 45 minutes before H/R. NC siRNA or Sab siRNA sequence was transfected into cells for 6 hours and then cultured with 10% FBS Opti-MEM™ medium for 48 hours. Also, 10 *μ*M PP2 was preincubated for 12 hours under normoxic conditions, and 10 *μ*M F_2_ was added to cells during prehypoxia for 30 minutes and hypoxia for 2 hours. Additionally, 20 ng/ml of Aniso was given to cells during hypoxia. The administration protocol was presented in Figure 1.

### 2.5. Cell Viability Assay

The viability of H9c2 cells was assessed by the MTT reagent according to the manufacturer's instructions [22]. H9c2 cells (5 × 10^3^) were seeded in 96-well plates for 48 hours and then subjected to hypoxia for 0, 1, 2, 4, 6, and 8 hours and reoxygenation for 1 hour. After the above treatment, 5 mg/ml MTT was added to the medium in each well, and the cells were incubated for 4 hours at 37°C with shielding from light. The medium was subsequently replaced with 150 *μ*l of dimethyl sulfoxide to solubilize the formazan. The absorbance was detected by a microplate reader (SpectraMax, Molecular Devices, Sunnyvale, CA, USA) at 490 nm.

### 2.6. Mitochondrial Isolation

Mitochondria were isolated by the method described by Wu et al. [23]. After the different treatments as indicated, the H9c2 cells were harvested. The mitochondria were isolated by using a mitochondria isolation kit. Briefly, cells were incubated in 500 *μ*l of the mitochondrial separation reagent containing 1 mM of phenylmethylsulfonyl fluoride and 1 mM of sodium orthovanadate for 15 minutes on ice and then were taken into a glass homogenizer and homogenized for 20 up-down strokes using a tight pestle on ice. The homogenate was separated by centrifugation at 1,000 g for 10 minutes at 4°C. The supernatant was collected and centrifuged again at 11,000 g for 15 minutes at 4°C to obtain the cytosolic (supernatant) and mitochondrial (deposition) fractions. Then, 76 *μ*l of mitochondrial lysis buffer was added into the isolated mitochondria. Finally, the protein concentration was measured by the bicinchoninic acid assay kit. Mitochondria were then homogenized for Western blot.

### 2.7. Western Blot Analysis

The protocol of extracting cells is as previously described [5, 20]. The equal denatured protein underwent 12% sodium dodecyl sulfate polyacrylamide gel electrophoresis and then was transferred to a nitrocellulose membrane by electrophoresis. Membranes were blocked with 5% fat-free milk and immunoblotted with diluted primary antibody: anti-phospho-JNK (1 : 400), anti-Sab (1 : 250), anti-p-Src (1 : 250), anti-c-Src (1 : 1000), anti-COX-IV (1 : 400), and anti-*β*-actin (1 : 5000) and then incubated at 4°C overnight, which was followed by exposure to horseradish peroxidase-conjugated secondary antibodies (typically 1 : 20,000 dilution) for 1 hour at room temperature. Transferred proteins were visualized by use of a SuperSignal detection kit (Thermo-Fisher Scientific, Waltham, MA, USA). The bands were analyzed with the Gel-Pro Image Analysis Software (Media Cybernetics, USA). The level of each mitochondrial protein was expressed as its density to the density of mitochondrial loading control COX-IV. To avoid the error in defferent batches, the desity ratio between interest protein and COX-IV in the H/R group was set as 1.

### 2.8. Immunofluorescent Detection of Colocalization of Phospho-JNK and Sab

H9c2 cells were seeded at glass coverslips placed in a 24-well dish. After being treated according to the different group protocols, the coverslips were washed with cold phosphate-buffered saline (PBS) and placed in 4% paraformaldehyde for 20 minutes at room temperature. After being permeabilized with 0.1% Triton X-100, the cells were blocked with goat serum for 1 hour at room temperature. The process described above is similar to our previous study [21]. Next, the cells were incubated with primary rabbit anti-phospho-JNK (1 : 50) and mouse anti-Sab (1 : 50) antibodies overnight at 4°C. Sab was detected using an Alexa Fluor™ 594-conjugated anti-mouse secondary antibody (Thermo-Fisher Scientific, Waltham, MA, USA), while phospho-JNK was detected using an Alexa Fluor™ 488-conjugated anti-rabbit antibody (Thermo-Fisher Scientific, Waltham, MA, USA). Finally, the cover glasses were mounted on a glass slide, and images were observed with a confocal laser scanning microscope (Carl Zeiss AG, Oberkochen, Germany).

### 2.9. Detection of Mitochondrial Superoxide

Mitochondrial superoxide production in H9c2 cells was detected with the mitochondria-selective dye MitoSOX™ Red (Thermo-Fisher Scientific, Waltham, MA, USA) [24] by using flow cytometry or the confocal laser scanning microscope. Briefly, cells were incubated with 5 *μ*M of MitoSOX™ Red (Thermo-Fisher Scientific, Waltham, MA, USA) for 10 minutes at 37°C in the dark. Then, cells were washed three times with PBS and monitored with flow cytometry at an excitation wavelength of 510 nm and an emission wavelength of 580 nm, and the image was recorded by the confocal laser scanning microscope and was analyzed by the Image-Pro Plus software (Media Cybernetics, US). The results were expressed as the fluorescence ratio of each group to the control group. The mean fluorescence intensity was analyzed using a BD Accuri™ C6 flow cytometer (BD Biosciences, San Jose, CA, USA).

### 2.10. Measurement of the Mitochondrial Nonyl Acridine Orange Level

The concentration of mitochondrial cardiolipin was presented by using nonyl acridine orange (NAO) [25]. For assessment of mitochondrial membrane damage, cells were stained with 100 nM of "NAO" at 37°C for 30 minutes in the dark. The cells were washed with PBS and analyzed immediately using the BD Accuri™ C6 flow cytometer (BD Biosciences, San Jose, CA, USA).

### 2.11. Measurement of Mitochondrial Membrane Potential

Mitochondrial membrane potential was monitored using the JC-1 dye kit [26]. H9c2 cells were seeded in a petri dish. After treatment, the cells were incubated with JC-1 for 20 minutes at 37°C in the dark, and images were taken with a fluorescence microscope (Olympus, Tokyo, Japan).

### 2.12. Manganese Superoxide Dismutase Activity

The activities of total SOD and copper and zinc SOD (CuZnSOD) in the H9c2 cells were measured by the colorimetric assay kit. The protocol used is as described in the manufacturer's instructions, and the details have been presented in our previous study [21]. Manganese SOD (MnSOD) activity = total SOD activity − CuZnSOD activity. All results were expressed as units per milligram of protein.

### 2.13. Statistical Analysis

The data are presented as mean ± standard error of the mean (SEM). Statistical significance was compared using one-way analysis of variance, followed by a Student–Newman–Keuls test, and *P* values less than 0.05 were considered to be statistically significant.

## 3. Results

### 3.1. Reactive Oxygen Species Levels and p-c-Jun N-Terminal Kinase Protein Expression in H9c2 Cell Cytoplasm and Cell Viability during Hypoxia/Reoxygenation over Time

**Figure 2:**
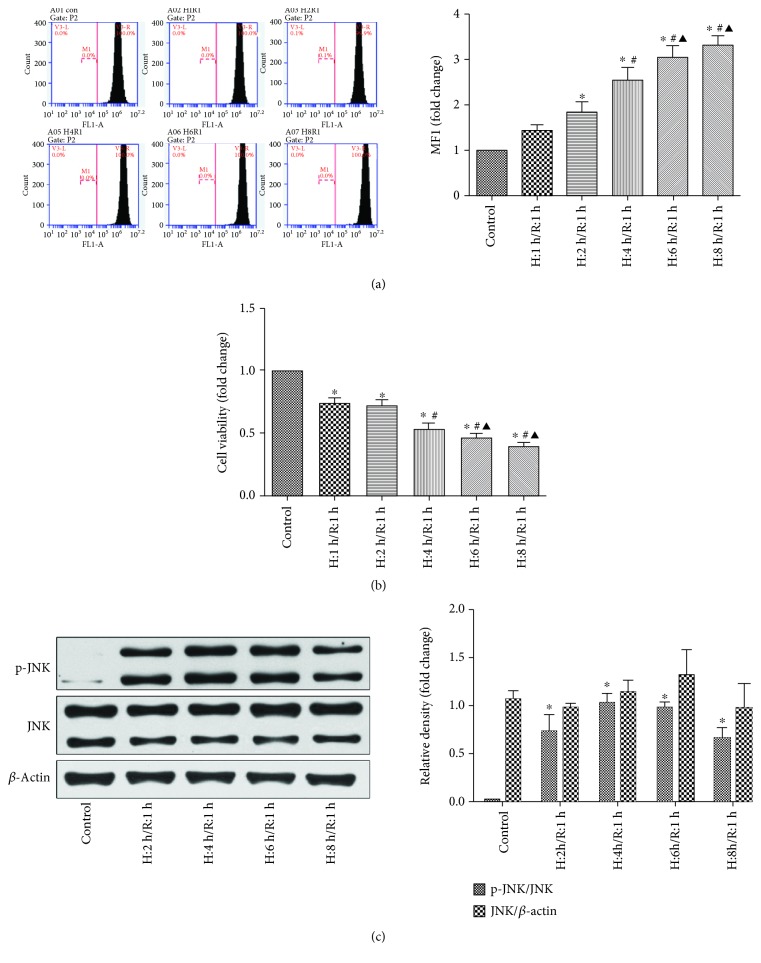
ROS levels and cell viability and JNK protein expression and activity in H9c2 cells following different durations of hypoxia and a 1-hour period of reperfusion. (a) ROS level measured by flow cytometry; *n* = 3. Data are expressed as the base of the levels of the control group. (b) Cell viability determined by the MTT assay; *n* = 3. Data are expressed as the base of the levels of the control group. (c) JNK and p-JNK protein levels as assessed by Western blot; *n* = 3. All values are represented as means ± SEMs. ^∗^*P* < 0.05 vs. control group; ^#^*P* < 0.05 vs. H: 1 hour/R: 1 hour group; ^▲^*P* < 0.05 vs. H: 2 hours/R: 1 hour group.

To investigate the effects of different durations of H/R on the ROS level and p-JNK expression in H9c2 cell cytoplasm and cell viability, H9c2 cells were cultured in a hypoxia/anaerobic workstation for 1, 2, 4, 6, or 8 hours and then returned to normal conditions for 1 hour of reoxygenation. Flow cytometry analysis revealed that H/R time-dependently increased ROS levels (Figure 2(a)), with a significant difference beginning at 2 hours of hypoxia and 1 hour of reoxygenation (H: 2 hours/R: 1 hour), respectively. Exposure of H9c2 cells to H/R resulted in a significant decline in cell viability with a time dependence (Figure 2(b)). We assessed the time course for JNK and p-JNK. JNK protein expression did not change in H/R over time as was expected (Figure 2(c)). In contrast, Figure 2(c) also shows H/R activated the phosphorylation of JNK as compared with the control group.

In comparison with the control group, the ROS level, JNK activity, and cell viability all remarkably changed beginning at H: 2 hours/R: 1 hour. Based on the above data, H: 2 hours/R: 1 hour were used in subsequent experiments.

### 3.2. Effects of c-Jun N-Terminal Kinase on Sab Protein Expression and Src Activity and the Reactive Oxygen Species Level in Mitochondria in H9c2 Cells

**Figure 3:**
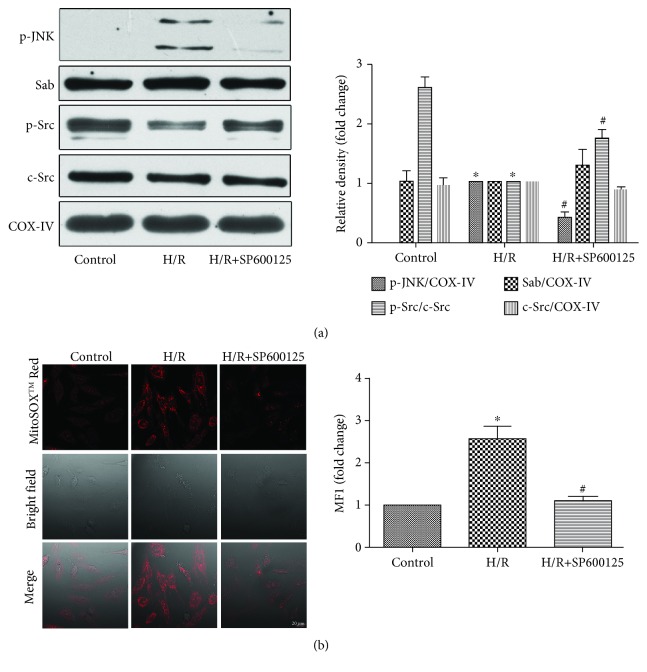
Effects of JNK on Sab protein and Src protein expression and the ROS level in mitochondria in H9c2 cells. (a) p-JNK, Sab, p-Src, c-Src, and COX-IV levels were analyzed by Western blot; *n* = 3. Data are expressed as the base of the levels of the H/R group. (b) The level of mitochondrial ROS was detected by the laser scanning confocal microscope, and the mean fluorescence intensity was measured by the Image-Pro Plus software; *n* = 3. Data are expressed as the base of the levels of the control group. All values are expressed as means ± SEMs. ^∗^*P* < 0.05 vs. control group; ^#^*P* < 0.05 vs. H/R group (×400, bar = 20 *μ*m).

To determine the expression of p-JNK in mitochondria during H/R and the effects of p-JNK on mitochondrial Sab and Src, we isolated mitochondria from H9c2 cells after treatment. As shown in Figure 3(a), there was no p-JNK localized to the mitochondria in the control group, but, after H/R treatment, p-JNK was found in the mitochondria and p-Src expression decreased. When JNK inhibitor SP600125 was used before H/R, the level of mitochondrial p-JNK markedly decreased and Src dephosphorylation was reversed. At the same time, the differences of Sab expression were not significant among each group (Figure 3(a)). Under normal conditions, the mitochondrial ROS level is lower. However, after H/R treatment, the mitochondrial ROS level increased, whereas SP600125 could decrease the level of mitochondrial ROS (Figure 3(b)).

### 3.3. Effects of Sab on p-c-Jun N-Terminal Kinase Protein Expression and Src Activity and the Reactive Oxygen Species Level in Mitochondria in H9c2 Cells

**Figure 4:**
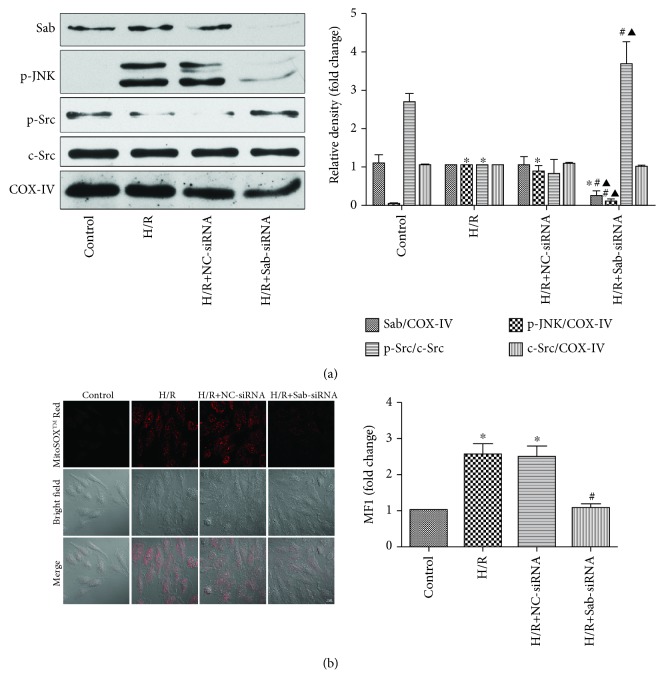
Sab mediates p-JNK translocation, Src activation, and mitochondrial ROS production in H9c2 cells subjected to H/R. (a) Sab, p-JNK, p-Src, c-Src, and COX-IV levels were analyzed by Western blot; *n* = 3. Data are expressed as the base of the levels of the H/R group. (b) The level of mitochondrial ROS was detected by the laser scanning confocal microscope; *n* = 3. Data are expressed as the base of the levels of the control group. All values are expressed as means ± SEMs. ^∗^*P* < 0.05 vs. control group; ^#^*P* < 0.05 vs. H/R group; ^▲^*P* < 0.05 vs. H/R+NC siRNA (×400, bar = 20 *μ*m).

We assessed the Sab dependence of the effects of JNK on mitochondrial Src activity by knocking down Sab expression using Sab siRNA.  Figure 4(a) shows that Sab siRNA successfully knocked down Sab expression. When we blocked Sab, mitochondrial p-JNK translocation was inhibited and Src dephosphorylation was reversed (Figure 4(a)). During the whole process, c-Src expression did not exhibit any change as we expected. The high level of mitochondrial ROS caused by H/R was inhibited when we knocked down Sab protein (Figure 4(b)). Additionally, as can be seen, NC siRNA had no effect on the expression of protein and the level of mitochondrial ROS.

### 3.4. Effects of Src Activity on Mitochondrial Reactive Oxygen Species Levels in H9c2 Cells

**Figure 5:**
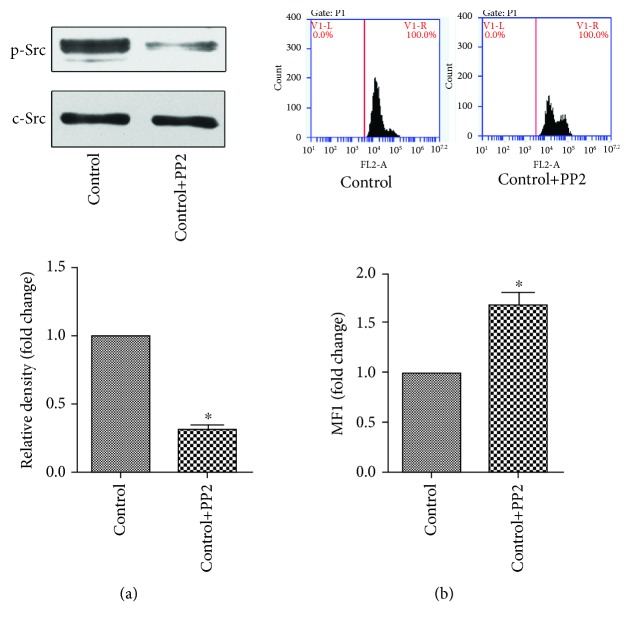
The effects of Src on the mitochondrial ROS level. (a) p-Src and Src protein expressions as measured by Western blot; *n* = 3. (b) Mitochondrial ROS level detected by flow cytometry; *n* = 3. Data are expressed as the base of the levels of the control group. All values are expressed as mean ± SEMs. ^∗^*P* < 0.05 vs. control group.

To further clarify the role of Src, we used the Src classical inhibitor PP2 to inhibit Src phosphorylation and observed its effect on mitochondrial ROS production (Figure 5(a)). Flow cytometry analysis indicated that inhibiting Src phosphorylation increased mitochondrial ROS levels (Figure 5(b)).

### 3.5. *N*-n-Butyl Haloperidol Iodide Inhibits the Mitochondrial JNK/Sab/Src/ROS Pathway in H9c2 Cells Subjected to Hypoxia/Reoxygenation

**Figure 6:**
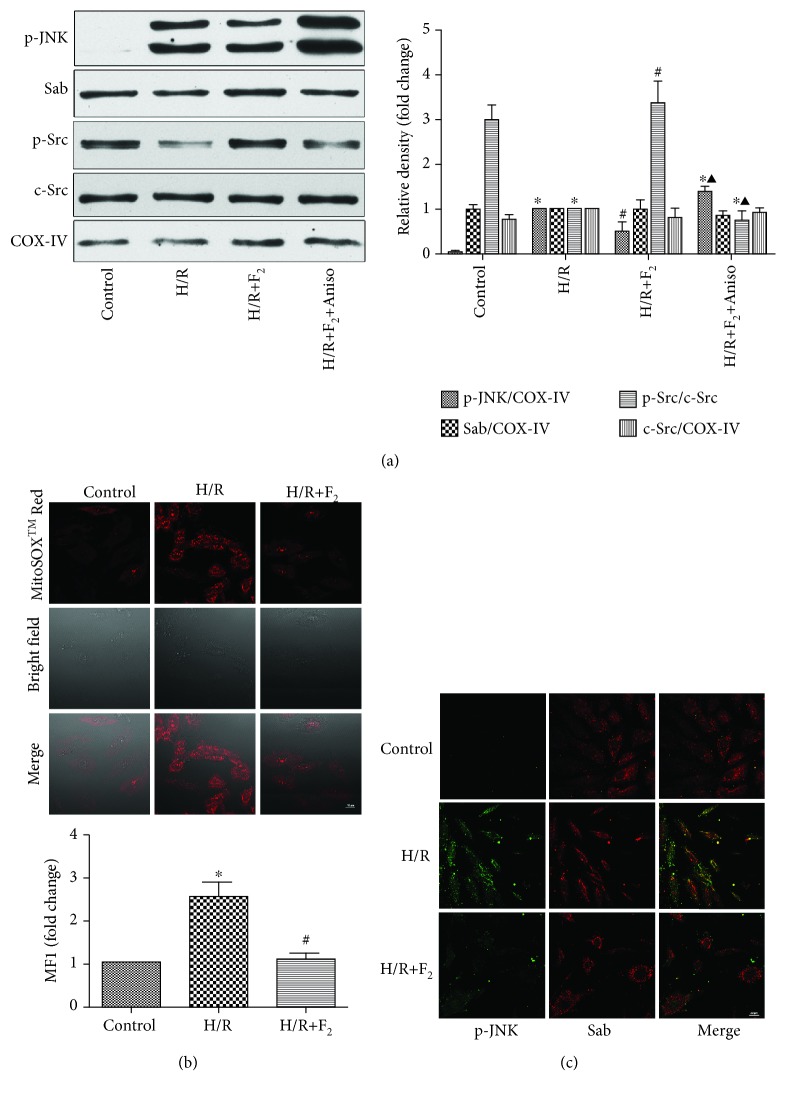
F_2_ regulates the mitochondrial JNK/Sab/Src/ROS pathway in the mitochondria of H9c2 cells following H/R. (a) p-JNK, Sab, p-Src, c-Src, and COX-IV levels were detected by Western blot; *n* = 3. Data are expressed as the base of the levels of the H/R group. (b) The effect of F_2_ on mitochondrial ROS generation was detected by the laser scanning confocal microscope; *n* = 3. Data are expressed as the base of the levels of the control group. (c) Colocalization of p-JNK and Sab in H9c2 cells was observed by the laser scanning confocal microscope. All values are expressed as means ± SEMs. ^∗^*P* < 0.05 vs. control group; ^#^*P* < 0.05 vs. H/R group; ^▲^*P* < 0.05 vs. H/R+F_2_ group (×400, bar = 20 *μ*m).

Based on the above-mentioned findings, we confirmed that there was a JNK/Sab/Src/ROS pathway in the mitochondria of H9c2 cells subjected to H/R. Next, we sought to ascertain the role of F_2_ in this pathway. As compared with the control group, both p-JNK and ROS production increased in the mitochondria of the H/R group, while p-Src expression decreased in the mitochondria of the H/R group (Figures 6(a)–6(c)). F_2_ could possibly reverse the above-mentioned change (Figures 6(a)–6(c)). However, JNK agonist Aniso also abrogated the F_2_-induced decrease in p-JNK expression and F_2_-induced increase in p-Src in H9c2 cells following H/R (Figure 6(a)). Still, there were no significant differences in Sab protein expression among all groups. To further verify the effects of F_2_ on mitochondrial p-JNK in a Sab dependence, immunofluorescence colocalization was used to explore whether there was interaction between p-JNK protein (green) and Sab protein (red) in H9c2 cells subjected to H/R and what the effects of F_2_ on them were. As illustrated in Figure 6(c), obviously, in the control group, p-JNK (green) is little; Sab (red) was found on the outer mitochondrial membrane. The treatment of H/R induced p-JNK generation and bound to Sab protein (yellow), but F_2_ blocked this interaction. The results showed that H/R caused the translocation of p-JNK into mitochondria and that F_2_ could inhibit this process.

### 3.6. The Relationship between Mitochondrial Oxidative Stress and the JNK/Sab/Src/ROS Pathway and the Effects of *N*-n-Butyl Haloperidol Iodide on Them

#### 3.6.1. Mitochondrial Membrane Potential

**Figure 7:**
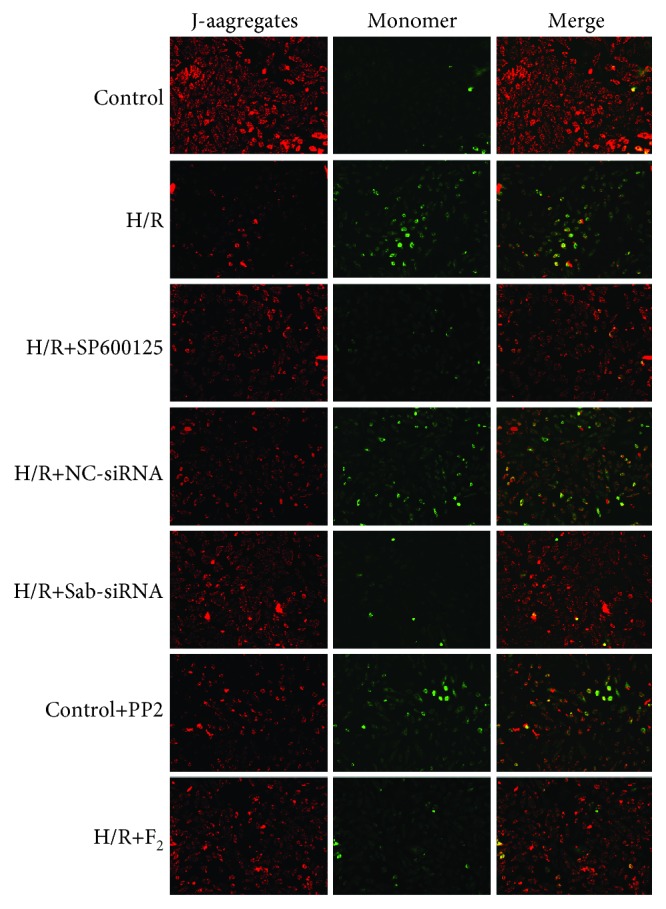
Mitochondrial membrane potential in H9c2 cells. The high-intensity red fluorescence indicates higher membrane potential, while the high-intensity green fluorescence signifies lower membrane potential (×100, bar = 20 *μ*m).

Via JC-1 staining, red fluorescence reflects higher mitochondrial membrane potential, and green fluorescence reflects lower mitochondrial membrane potential. As illustrated in Figure 7, red fluorescence was stronger in the control group, which indicated a higher mitochondrial membrane potential in normal conditions. As compared with the control group, the H/R group, the H/R+NC siRNA group, and the control+PP2 group all had weaker red fluorescence and stronger green fluorescence, which indicated mitochondrial damage. However, the treatment of Sab siRNA, SP600125, and F_2_ alleviated H/R-induced lower mitochondrial membrane potential. These findings suggest that inhibiting the JNK/Sab/Src pathway or using F_2_ could increase mitochondrial membrane potential and ameliorate mitochondrial injury.

#### 3.6.2. Manganese Superoxide Dismutase Activity

MnSOD activity was examined to assess the degree of mitochondrial antioxidative ability in H9c2 cells subjected to H/R. In comparison with the control group, the treatment of H/R and PP2 decreased MnSOD activity. In contrast, using SP600125, Sab siRNA, or F_2_ could reverse H/R-induced lower MnSOD activity (Table 2).

#### 3.6.3. Mitochondrial Nonyl Acridine Orange Content

**Figure 8:**
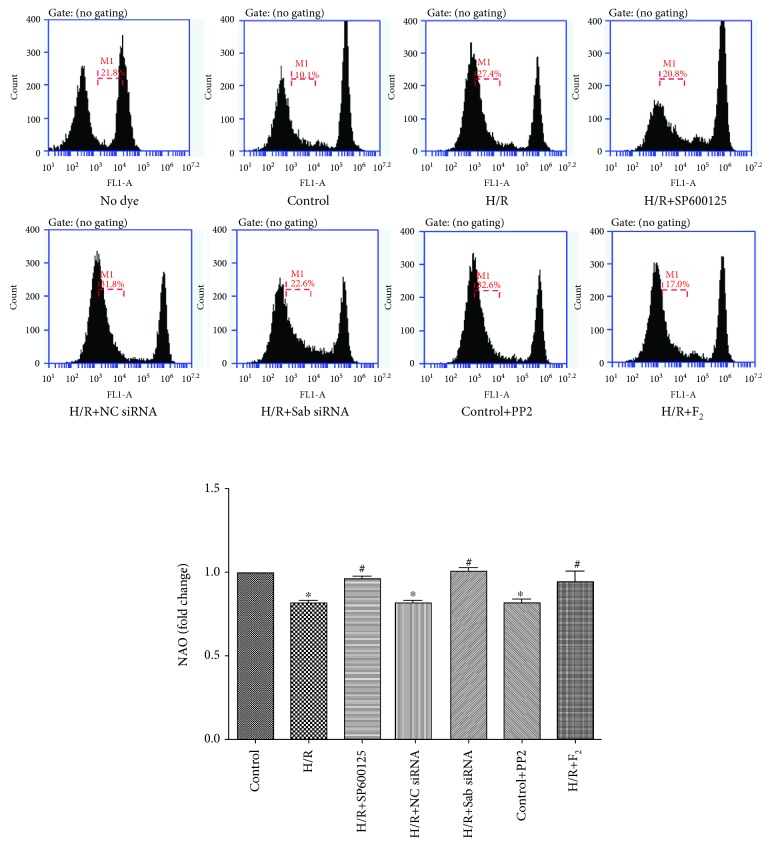
NAO levels in the mitochondria of H9c2 cells as measured by flow cytometry. Data are expressed as the base of the levels of the control group. All values are expressed as mean ± SEMs; *n* = 3. ^∗^*P* < 0.05 vs. control group; ^#^*P* < 0.05 vs. H/R group.

To further confirm the degree of mitochondrial oxidative stress damage, NAO fluorescence dye was used to evaluate the oxidative damage of the mitochondrial cardiolipin membrane. As shown in Figure 8, the treatment of SP600125, efficient knockdown of Sab, and use of F_2_ improved the H/R-induced oxidative damage of cardiolipin membrane, whereas inhibiting Src phosphorylation by PP2 triggered mitochondrial oxidative stress.

## 4. Discussion

Mitochondria in cardiomyocytes contribute to but in reverse suffer from oxidative stress injury. Damaged mitochondria not only cannot provide enough energy to the myocardium but also initiate additional myocardial I/R damage through other mechanisms, such as inducing apoptosis by releasing cytochrome C and an apoptosis-inducing factor [27–29], influencing the calcium balance of cardiomyocytes, disrupting the many signal chains of calcium ion downstream, and ultimately causing myocardial contractility dysfunction. The results of our laboratory study on primary cardiomyocytes or H9c2 cells showed that H/R causes mitochondrial swelling and crest reduction or disappearance and cellular apoptosis [20]. The above findings confirmed that H/R can cause mitochondrial damage and can cause whole-cell damage. In the present study, our results showed that the H/R stimulates mitochondrial ROS generation. At the same time, mitochondrial membrane potential, NAO levels, and MnSOD levels were also used to reflect mitochondrial damage, cardiolipin membrane oxidation, and mitochondrial SOD-scavenging ability, respectively. The results also showed that mitochondrial membrane potential, MnSOD activity, and NAO levels decreased after H/R in H9c2 cells, indicating that there were too many ROS produced in the mitochondria to clear, which resulted in oxidation of the cardiolipin membrane and subsequent mitochondrial oxidative stress injury. This injury initiated the prelude to comprehensive myocardial I/R injury.

JNK is a member of the MAPK family, which modulates various cellular functions, including proliferation, differentiation, and cell apoptosis [30]. Research on H/R or I/R models in the myocardium [31], renal cells [32], and the brain [33] showed that activated JNK mediates injury and that inhibition of p-JNK expression can reduce H/R- or I/R-induced apoptosis, death, or necrosis. Previous studies in our laboratory also indicated that the inhibition of JNK activation can improve the survival of cardiac microvascular endothelial cells [5], which were supported by the results of Li et al. [31] and Sun et al. [34]. Our present study also found that the JNK inhibitor SP600125 improves H/R-induced H9c2 cell viability (data not shown), suggesting that p-JNK is an injury factor in this experimental pathology model. In comparison with the control group, JNK activities were significantly higher in the cytoplasm of the H9c2 cells of each H/R group. Although the expression of p-JNK decreased slightly after H: 6 hours/R: 1 hour, it is still at a very high level in comparison with the control group. This trend of variation is similar to the results of Chambers et al. [17] obtained using a rat myocardial I/R model, which suggests that the model of H9c2 cell H/R injury established by the hypoxic/anaerobic workstation is very similar to the in vivo model of myocardial I/R injury.

Recently, Win et al. [16] proposed that cytoplasmic p-JNK can bind to the mitochondrial outer membrane Sab protein, resulting in mitochondrial Src inactivation and further inhibiting the electron transfer chain, which increases mitochondrial ROS generation and causes liver damage. A study involving an H_2_O_2_/FeSO_4_-induced H9c2 cell oxidative stress injury model showed translocation of p-JNK into the mitochondria and Sab binding, causing cell damage. When p-JNK translocation to the mitochondria was inhibited or its binding to the Sab protein was blocked, there was less cell damage [17]. Wiltshire et al. [35] first reported that p-JNK could bind to the Sab protein in the mitochondria, which provided the theoretical bases for the effect of p-JNK on mitochondria. In addition, a study has suggested that Src is necessary to maintain respiratory chain activity [36]. Ge et al. [15] found that the mitochondrial Src activated by ischemic preconditioning could regulate the activity of complex I and the level of mitochondrial ROS to antagonize myocardial I/R injury. It has not yet been elucidated whether abundantly activated JNK in the cytoplasm will translocate to mitochondria and what the relationship is between p-JNK and Sab, Src, and ROS in the mitochondria during myocardial I/R. Our results showed that, in H9c2 cells, H/R stimulated mitochondrial p-JNK expression and Src dephosphorylation but had no effect on the expression of Sab protein. In order to verify the existence of the interaction between p-JNK and Sab in H9c2 cells, an immunofluorescence colocalization technique was used to identify the p-JNK and Sab proteins. The results showed that the overexpression of p-JNK caused by H/R significantly increased the possibility of interaction with Sab protein in mitochondria, and Western blot results also showed that Sab siRNA significantly reduced the amount of p-JNK localized in the mitochondria. Taken together, the above results indicate that the mitochondrial translocation of cytoplasmic p-JNK is mediated by the anchoring protein Sab on the mitochondrial membrane during H/R stimulation of H9c2 cells. The results also showed that inhibiting the expression of p-JNK in mitochondria reduced the degree of dephosphorylation of Src, decreased the production of ROS in the mitochondria, and mitigated oxidative stress in the mitochondria, as evidenced by the increase in MnSOD activity, NAO levels, and mitochondrial membrane potential. While inhibiting Src phosphorylation that resulted in rapid generation of ROS in the mitochondria, a decrease in MnSOD activity, NAO levels, and mitochondrial membrane potential also occurred. These results support the hypothesis that there is a JNK/Sab/Src/ROS signal pathway in the mitochondria of H9c2 cells under H/R, which is closely related to mitochondrial oxidative stress injury. Inhibition of this pathway during H/R could reduce mitochondrial oxidative stress damage. Our previous study showed that ROS production during the process of H/R could increase the activation of cytoplasmic JNK [12], indicating that ROS can regulate the activation of cytoplasmic JNK during H/R. This study showed that inhibition of JNK activation in the cytoplasm will reduce mitochondrial translocation of p-JNK and ultimately lower ROS production in the mitochondria. Our results are in agreement with the study of Chambers et al., who found that H_2_O_2_/FeSO_4_-stimulated H9c2 cells increase the expression of p-JNK and induce ROS accumulation in the mitochondria [37]. On the basis of our results in combination with the literature, we propose that there is an ROS-induced ROS release loop in the myocardium following I/R—that is, an ROS/JNK/ROS loop mediated by Sab and Src proteins.

**Figure 9:**
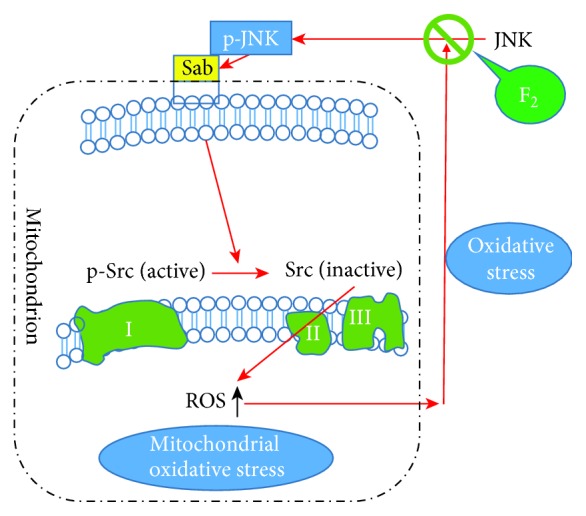
Model of the JNK/Sab/Src/ROS signaling pathway and the role of F_2_ in H9c2 cells during H/R. H/R causes oxidative stress in mitochondria by activating JNK, which binds to mitochondrial outer membrane Sab protein, leading to inactivation of mitochondrial p-Src and increasing mitochondrial ROS production in H9c2 cells. F_2_ could inhibit H/R-induced JNK activation and ameliorate dephosphorylation of Src and reduce the mitochondrial ROS level.

In our previous research, we also found that F_2_ could improve mitochondrial swelling and crest reduction or disappearance induced by H/R, mitigate mitochondrial damage, and antagonize the increase of cleaved caspase-3 induced by H/R, as well as inhibit the release of cytochrome C from the mitochondria [20] and apoptosis [38]. In addition, F_2_ inhibits I/R- or H/R-induced MDA production, an important index of ROS-induced lipid peroxidation, in the whole heart [39], cardiac-derived cardiomyocytes [6], and cardiac-derived microvascular endothelial cells [40]. At the same time, it exhibits an inhibitory effect on ROS-induced cardiac I/R- or H/R-associated inflammation as evidenced by the amelioration of Egr-1 overexpression. Therefore, F_2_ has a multiprotective effect on myocardial I/R injury. Via in-depth study on the mechanism of F_2_, we observed that it can regulate the abnormal ROS/JNK signaling pathway in the cardiomyocytes experiencing H/R and cardiac microvascular endothelial cells and antagonize whole-cell oxidative stress injury, including cell lipid membrane oxidation and cytosolic total SOD activity [5, 12]. In the present study, we further observed that F_2_ could inhibit the H/R-induced mitochondrial translocation of p-JNK and reduce the binding of p-JNK to the mitochondrial Sab protein, thereby inhibiting the dephosphorylation of Src by Sab protein and ROS generation, reducing mitochondrial oxidative stress, as demonstrated by the increasing MnSOD activity, NAO levels, and mitochondrial membrane potential. Altogether, this study demonstrated that F_2_ can reduce the damage associated with H/R-induced mitochondrial oxidative stress by modulating JNK/Sab/Src/ROS signaling pathways (Figure 9). At the same time, combined with previous research conducted by our group, we inferred that the effect of F_2_ on protecting cells from H/R-induced injury is related to adjusting the ROS/JNK/Sab/Src/ROS loop.

That being said, for now, we have no direct evidence that F_2_ derived from haloperidol still retains *α*1 adrenergic receptor blocking property, which results in its inhibition to ROS, but our previous studies suggest that myocardial I/R causes the decrease in systolic blood pressure (SP), diastolic blood pressure (DP), and mean arterial pressure (AP) in rats, respectively, whereas the administration of F_2_ reverses the decline of SP, DP, and AP induced by I/R [41], indicating F_2_ has no obvious effect to the *α*1 adrenergic receptor on peripheral vasodilation. Nevertheless, it is not clear whether F_2_ can block the *α*1 adrenergic receptor on the heart and thus affect ROS production, which will be the next interesting research direction.

## 5. Conclusions

In summary, H/R leads to the activation of a JNK/Sab/Src/ROS signaling pathway in the mitochondria of H9c2 cells, resulting in mitochondrial oxidative stress. F_2_ ameliorates H/R-induced mitochondrial oxidative stress through inhibiting the activation of the JNK/Sab/Src/ROS signaling pathway induced by H/R.

## Figures and Tables

**Table 1:** siRNA duplexes.

RAT-Sh3bp5-siRNA-1	Sense	5′- GAAAUGCUGAAUCACGCUAdTdT -3′
	Antisense	5′- UAGCGUGAUUCAGCAUUUCdTdT -3′

RAT-Sh3bp5-siRNA-2	Sense	5′- GUUAAAUCAAUCCACCGAUdTdT -3′
	Antisense	5′- AUCGGUGGAUUGAUUUAACdTdT -3′

RAT-Sh3bp5-siRNA-3	Sense	5′- GGAUCUCGGAUGAGAUACAdTdT -3′
	Antisense	5′- UGUAUCUCAUCCGAGAUCCdTdT -3′

Negative control	Sense	CGUUUGUUCGCUUCCUGAGTT
	Antisense	CUCAGGAAGCGAACAAACGTG

**Table 2:** Effects of F_2_ and inhibitors of the JNK/Sab/Src signal pathway on the change of MnSOD activity stimulated by H/R in H9c2 cells (mean ± SEM, *n* = 6).

Group	MnSOD (U/mg prot)
Control	8.795 ± 0.7465
H/R	2.003 ± 0.5865^∗^
H/R+SP600125	8.096 ± 0.7342^**#**^
H/R+NC siRNA	0.2478 ± 1.386^∗^
H/R+Sab siRNA	7.443 ± 2.262^#^
Control+PP2	2.149 ± 0.7925^∗^
H/R+F_2_	8.701 ± 0.7400^**#**^

^∗^ *P* < 0.05 vs. control group; ^#^*P* < 0.05 vs. H/R group.

## Data Availability

The data used to support the findings of this study are available from the corresponding author upon request.
